# Self-powered Real-time Movement Monitoring Sensor Using Triboelectric Nanogenerator Technology

**DOI:** 10.1038/s41598-017-10990-y

**Published:** 2017-09-05

**Authors:** Liangmin Jin, Juan Tao, Rongrong Bao, Li Sun, Caofeng Pan

**Affiliations:** 1 School of Information & Computer, Anhui Agricultural University, Hefei, 230036 China; 2 Beijing Institute of Nanoenergy and Nanosystems, Chinese Academy of Sciences; National Center for Nanoscience and Technology (NCNST), Beijing, 100083 China

## Abstract

The triboelectric nanogenerator (TENG) has great potential in the field of self-powered sensor fabrication. Recently, smart electronic devices and movement monitoring sensors have attracted the attention of scientists because of their application in the field of artificial intelligence. In this article, a TENG finger movement monitoring, self-powered sensor has been designed and analysed. Under finger movements, the TENG realizes the contact and separation to convert the mechanical energy into electrical signal. A pulse output current of 7.8 μA is generated by the bending and straightening motions of the artificial finger. The optimal output power can be realized when the external resistance is approximately 30 MΩ. The random motions of the finger are detected by the system with multiple TENG sensors in series. This type of flexible and self-powered sensor has potential applications in artificial intelligence and robot manufacturing.

## Introduction

With the rapid development of the economy and improvement in the standard of living, an eagerness for smart electronic devices capable of being flexible, miniature, and stretchable, such as human–machine interactions, artificial electronic skin, and sensors system, has increased^1–3^. Most of these devices are powered by batteries with the disadvantages of large-size, heavy-weight, and limited lifetime, hindering the modern designed conception of mobility and sustainability. Thus, the reliable and sustainable power resource supplying these portable devices or sensors remains as one of the most crucial issues. In addition, renewable and clean energy, such as wind, solar energy, and mechanical energy, are urgently being pursued due to the growing crisis of energy shortage and the deterioration of the environment^4, 5^. Distinguished from others, the ambient mechanical energy of daily life, such as from sound, ambient vibration, and human body movements, is one of the most general sources of energy, which can be everywhere and at any time^6, 7^.

Recently, a new type of technology, the triboelectric nanogenerator (TENG), has been also demonstrated to establish a platform for developing self-powered sensor systems and flexible/wearable electronics to harvest mechanical energy from the surrounding environment into electricity^8–10^. The working principle of the TENG is a coupling of the triboelectric effect and electrostatic induction, and charge transfer occurs when two different frictional materials with opposite triboelectric polarities come in contact with each other, generating opposite and equivalent charges at the interface^11–13^. Compared with traditional technology, TENG has many advantages, including large power density, high efficiency, low-cost, simple device fabrication, and abundant choice of materials^14–17^. Thus, it has been demonstrated in various applications, such as powering commercial LEDs^18, 19^, charging capacitors^20^, driving an electronic watch or calculator^12, 21^, protecting metal from corrosion^20, 22^, and temperature sensor^23^.

Meanwhile, there has been growing interest in human-motion wearable sensors, especially in high-impact applications such as activity recognition and health sensing^24, 25^, many of which have power consumption. Tremendous efforts have been paid to reduce energy consumption and investigate the new-type motion sensor with high stretchability, stability and fast response. For instance, static electric fields is used to render an ultra-low-power method for passively sensing body motion only 3.3 μw^26^; aligned single-walled carbon nanotube/polymer has been adopted to detect strains up to 280% and deferent types of human motion^27^, and a silver nanoparticle thin film patterned on the polydimethylsiloxane (PDMS) is utilized as the strain sensor to detect tensile and compressive strains^28^. In this paper, TENGs combined with an artificial finger was chosen to act as motion sensor to detect finger movement without any additional power support. At the same time, there exists energy conversion from mechanical energy of finger movement to electricity with the help of TENG. The transformational electricity provides a great potential in supplying other detecting sensors and wearable electronic devices, which would benefiting the integration of smart electronic devices.

Herein, we present a TENG combined with an artificial finger as a self-powered sensor system as a potential approach for the real-time monitoring of finger movements without any other external power sources. Under the movement of a finger, the TENG realizes the contact and separation to transform the mechanical energy into electrical signal. First, a single TENG is designed to investigate its basic properties. A pulse output current of 7.8 μA is generated with finger bending and straightening motions. The transferred charge quantity and voltage, which are independent of frequency, can reach 78 nC and 130 V, respectively, in a complete process. Then, the optimal output power can be realized when the external resistance is approximately 30 MΩ. Finally, a self-powered sensor system is fabricated by the TENGs attached to different joints to detect and monitor the random motions of the finger. The transferred charge quantity is chosen as a characteristic parameter. This work establishes a self-power monitoring system to detect the real-time motion of a finger by scavenging its mechanical energy, which largely enriches the application of the TENG and provides a potential in other detecting applications.

## Results and Discussion

**Figure 1:**
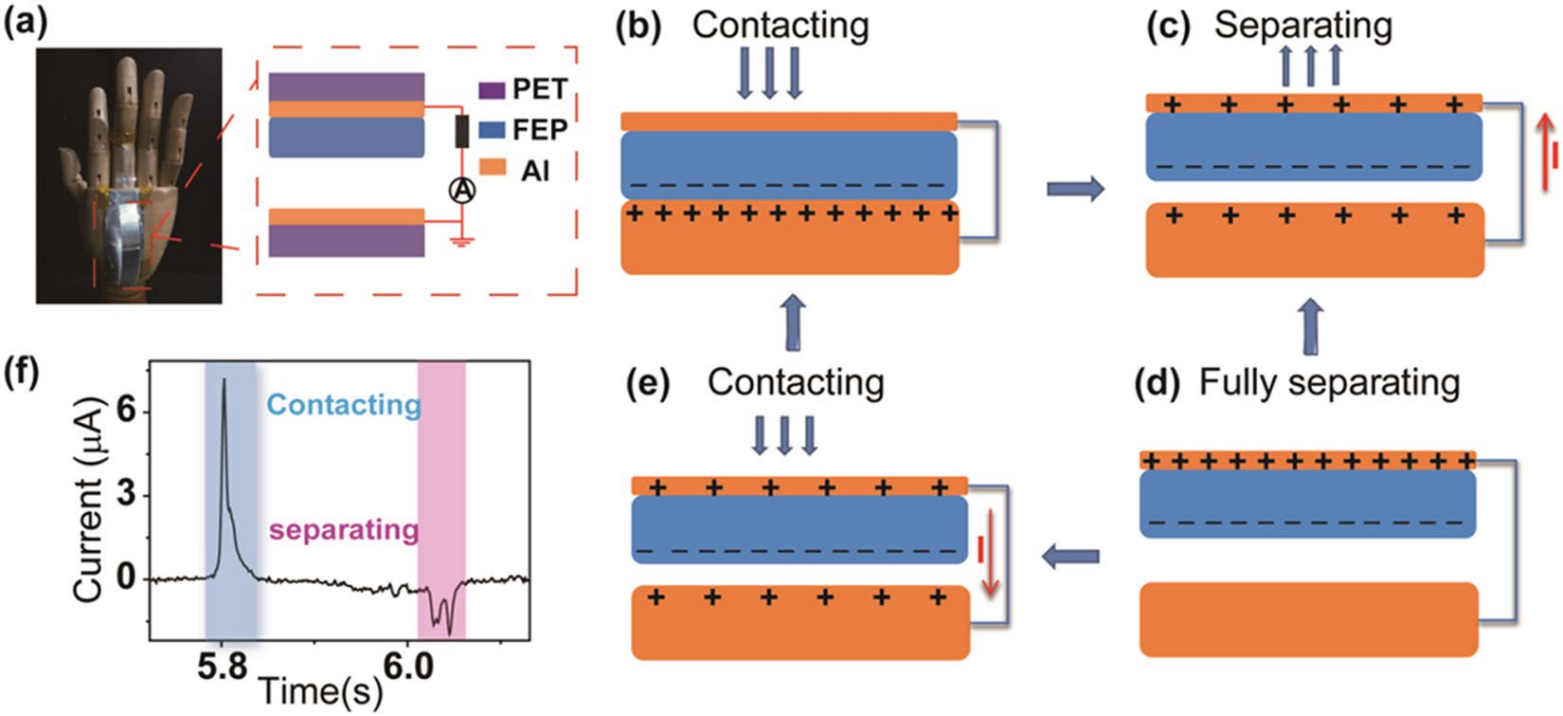
(**a**) A digital photograph and schematic diagram of the TENG combined with the artificial hand. The bending and straightening movement of the finger causes contacting and separating states of the TENG. (**b**–**e**) The working principle of the TENG in four steps. (**f**) The reversed current signal in one process of contacting and separating.

The basic unit of the TENG combined with the finger has two segments, as schematically shown in Fig. 1a. One part is adhered to the back of the hand acting as a stationary plate. The other can be bent or unbent, depending on the movement of the finger, with one end fixed to the back of hand and the other end adhered to the finger. The substrates of both are made of polyethylene terephthalate, which will accomplish contacting and separating actions with the help of its natural flexibility. As seen in the magnified structure schematic, aluminium film is chosen for the electrodes, and a 30 µm FEP film acts as one of the frictional materials. For the fixed part, the Al film acts as both electrodes and frictional material. Since FEP has a higher triboelectric negative series than aluminium, electrons from aluminium will be injected onto the FEP. When the bending action of the finger triggers contact of the FEP and aluminium (Fig. 1b), positive charges on the aluminium surface and negative ones on the FEP surface generate due to the contact electrification effect. Subsequently, if the finger begins to straighten, the elasticity will lead to a separation of the FEP and aluminium (Fig. 1c). Therefore, a potential drop is created between the two electrodes, which will drive the free electrons move from the back electrode of the FEP to another aluminium electrode through an external circuit to balance the electric field, yielding a positive charge net on the back aluminium electrode of the FEP. The transferred charges will not achieve a maximum value until the distance reaches its highest point, which corresponds to the maximized separation (Fig. 1d). The decrease in distance between the FEP and aluminium will weaken the potential difference, which induces electrons flow inversely to maintain the balanced state until the two surfaces come into contact, causing a reversed current output (Fig. 1e). Thus, the whole cycle of the electricity generation process contains contacting and separating. As a consequence, an inversed pulse current signal is produced (Fig. 1f). Furthermore, a higher and narrower amplitude electricity in the contacting signal and a lower and wider amplitude current signal in the separating process can be observed. However, the integrat areas of the two currents are nearly the same. The reason for the above phenomena lies in a faster speed of the bending operation than the straightening action of the finger. It is worth noting that there exist two peaks in the operation of separation, which may be caused by the back of the hand slightly curving, resulting in a moderately incomplete separation process.

**Figure 2:**
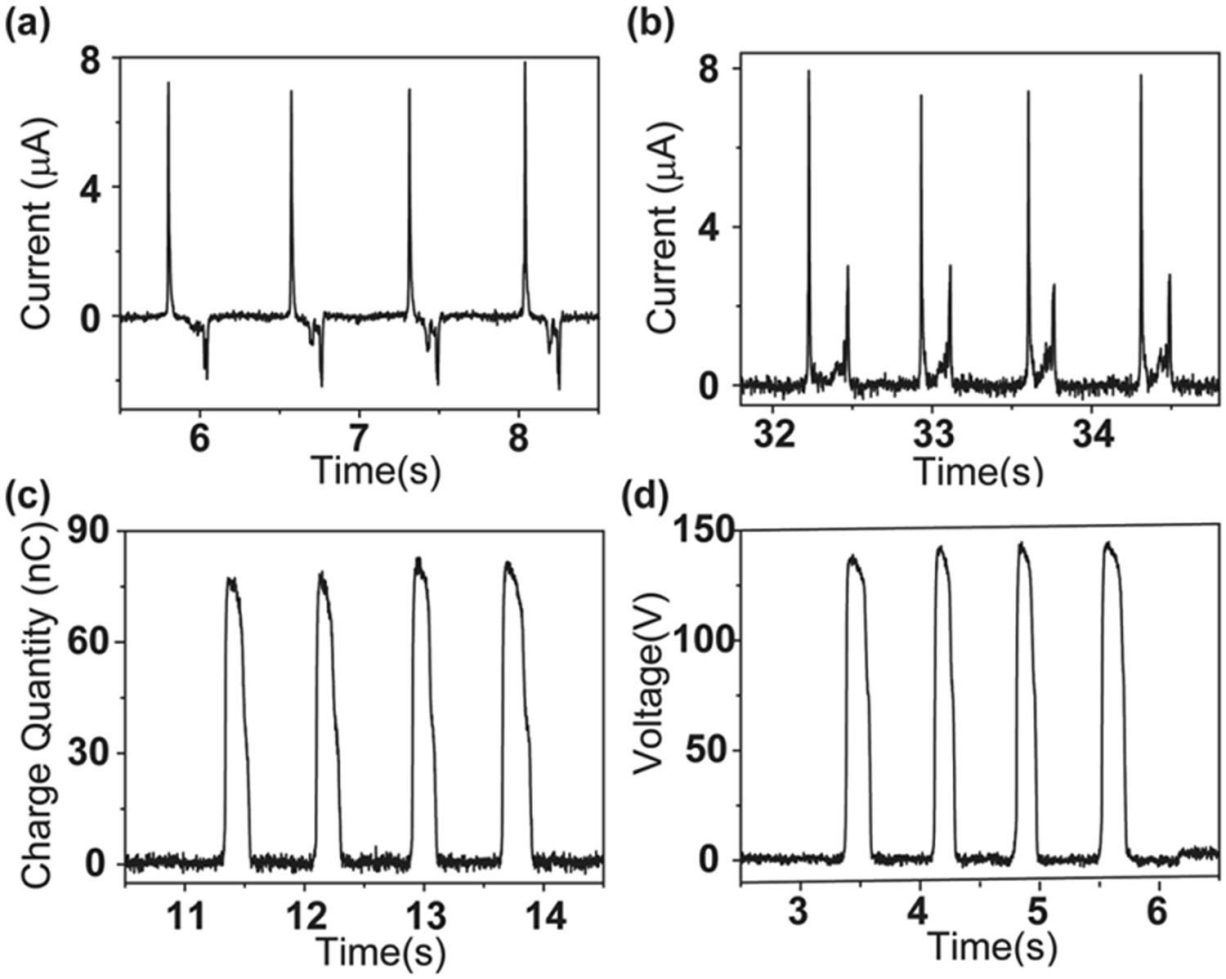
Electric characteristics of the TENG. (**a**–**d**) The measurement results of the short-circuit current, rectified short-circuit current, transferred charges and voltage of the TENG, respectively.

The electrical output characteristics of the TENG unit are measured and shown in Fig. 2. The measurement is carried out in the operation by manually. In addition, all output tests are performed under the same conditions with the nearly uniform speed of manual operation. The short circuit current and rectified current are respectively exhibited in Fig. 2a,b. It is apparent that the TENG has a high output performance as the current and rectified current signals are approximately 7.8 µA. And the transferred charge quantity and the voltage value can reach 80 nC and 130 V, as displayed in Fig. 2c,d. The above electric output can be used in various self-powered system applications, such as for driving several commercial LEDs, charging a capacitor, corrosion protection, and powering an electronic calculator or timer for a long time.

**Figure 3:**
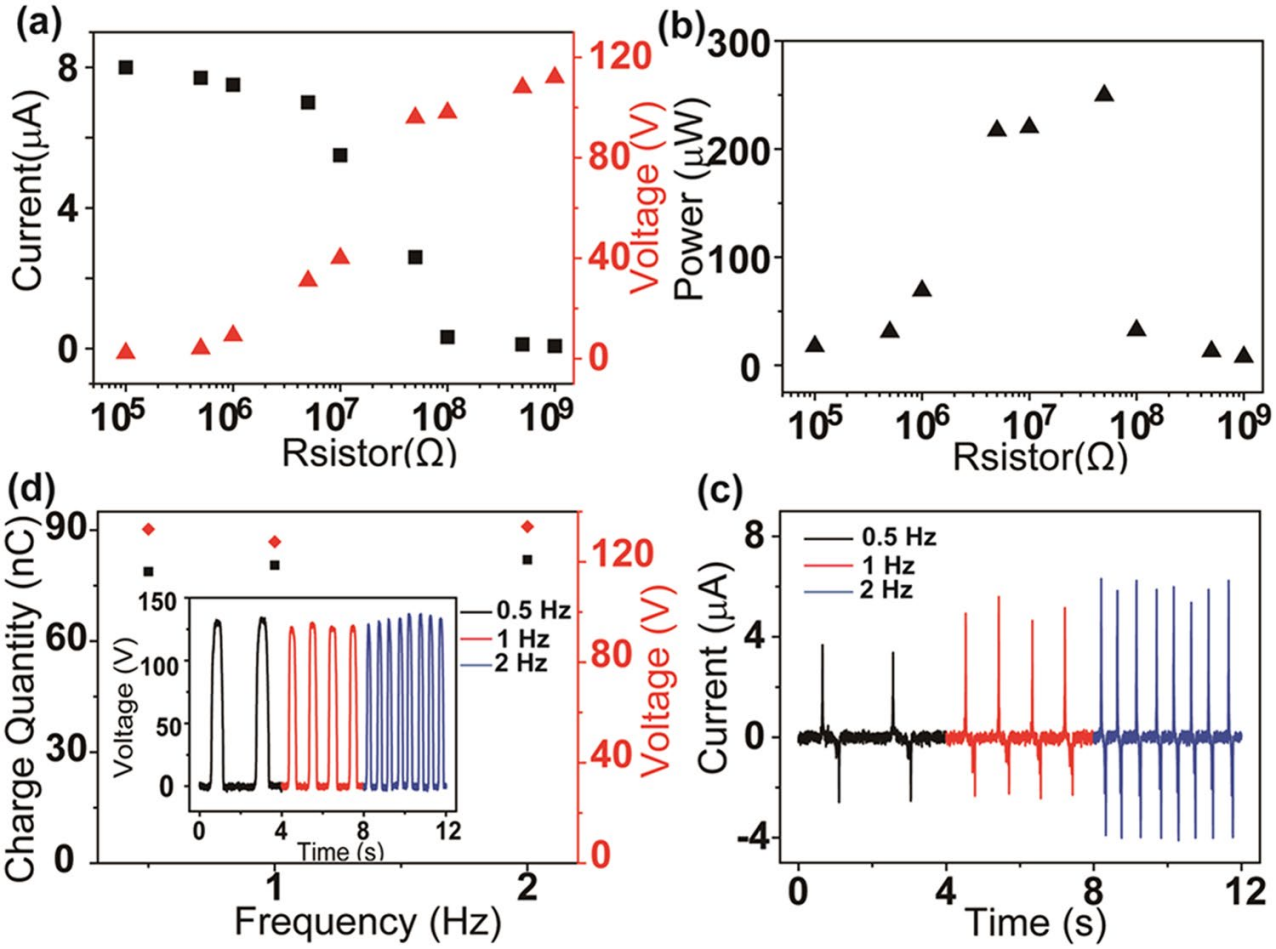
(**a**) Output current and voltage under variable load resistance. (**b**) Output power under different load resistances. (**c**) Charge quantity and voltage amplitude with frequencies of 0.5 Hz, 1 Hz, and 2 Hz (inset: the accurate voltage amplitude at different frequencies). (**d**) Current amplitude at frequencies of 0.5 Hz, 1 Hz, and 2 Hz.

To clearly discover the properties of the TENG combined with a hand, different resistors are utilized as external loads to study the output power under the same condition. As depicted in Fig. 3a, the measured voltage and current outputs of the TENG present an inversed tendency. The current amplitude reduces while the voltage increases with the increase of the load resistance, owing to the ohmic loss. Below 1 MΩ, the current decreases slightly, and the voltage remains almost zero. Once the resistance is larger than 1 MΩ, the current will decrease rapidly, and the output voltage will increase sharply. Therefore, there exists an optimal resistive load and optimum output power. As displayed in Fig. 3b, when the maximum instantaneous power generated by the TENG reaches approximately 250 μW, the optimal resistance is at approximately 30 MΩ. Moreover, the dependence of the open circuit voltage, charge quantity and short circuit current on the operational frequency of the finger are respectively exhibited in Fig. 3c,d. According to the results, with the increase of the working frequency from 0.5 to 2 Hz, the transferred charges and the voltage do not experience any meaningful change, remaining at approximately 78 nC and 130 V, respectively. However, the current amplitude enhances from 3.8, 5 to 6.2 µA as the frequency increases from 0.5, 1 to 2 Hz (Fig. 3d), respectively. This is because both the voltage and transferred charge are mainly determined by the structures or the frictional materials in the TENG, while at a higher frequencies, more charge transfer cycles occur in a given period of time, yielding a higher output current.

**Figure 4:**
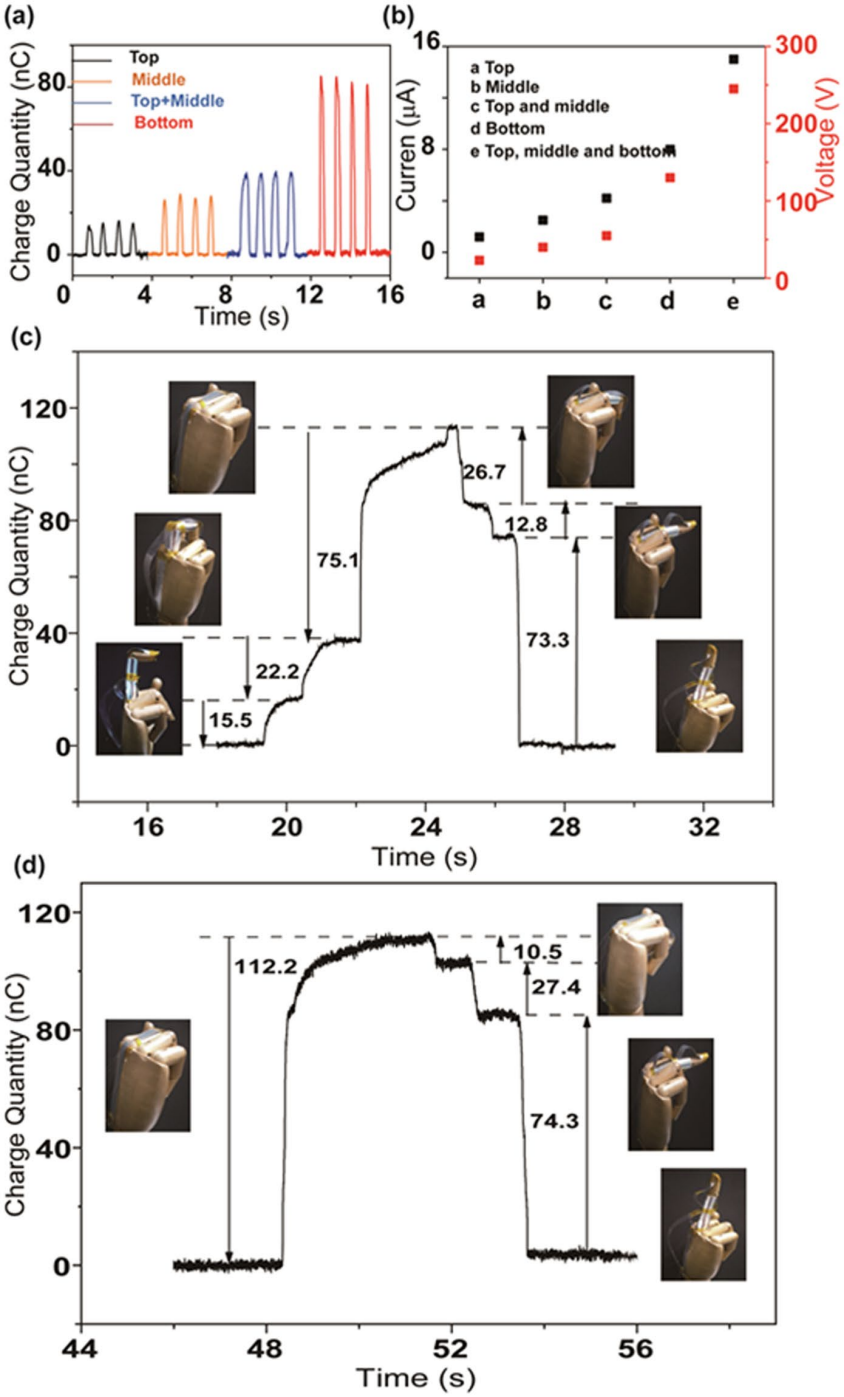
(**a**,**b**) Transferred charge quantity, current, and voltage signal when the top, middle, top and middle, and bottom joint complete a bending and unbending process. (**c**,**d**) Transferred charge quantity curve of two different random movements of the middle finger.

Since the TENG can harvest mechanical energy from the movement of the finger and convert it into electricity signals, more TENGs have been designed to be combined with different joints to realize a self-powered sensor system. The middle finger, for example, has TENGs with same structures as above for the top, middle and bottom joint. For the top joint sensor, the related TENG is mainly assembled in the middle joint with the mobile end adhered to the top. Thus, the bending and unbending action of the top joint will trigger the contacting and separating process of the TENG. According to this law, the middle and bottom joints related to the TENGs should be set in the bottom joint and the back of the hand, respectively. The area of each TENG differs from the other at 3 cm^2^, 4.5 cm^2^ and 10.5 cm^2^. As illustrated in Fig. 4a, the transferred charges present an increasing tendency, where the value of the top, middle, top and middle, and bottom are approximately 15 nC, 25 nC, 40 nC, and 78 nC, respectively, over the entire bending and unbending operation. The greater transferred charge quantity is attributed to the larger contact area. Additionally, the measured current and voltage output show a similar trend, as shown in Fig. 4b. It is apparent that the current and the voltage have significantly increased. According to the experimental results, the transferred charges remain relatively stable and are independent of the frequency compared with the current signal in a complete cycle. Therefore, the transferred charge quantity of the TENGs from different joints is chosen as the evaluation index in the self-powered sensor system to eliminate the uncontrollable manual operation. If the middle finger has random motions under the manual manipulation, the specific process of any joint can be deduced according to the transferred charge curves.

Two complicated and random examples are depicted in Fig. 4c,d, respectively. As seen in Fig. 4c, the transferred charge curve initially has three rising steps until the highest point and three subsequent declining steps. The first increasing step has an amplitude of 15.5 nC, consistent with the bending action of the top joint based on the characteristic parameter and magnitude sequence of the various joints. Thus, the following actions of the rising process are deduced as 22.2 nC and 75.1 nC in line with the bending of the middle and bottom joints in order. Until to the highest point, the entire middle finger is completely in a bent state. In the declining trend, the value of the transferred charges of 26.7, 12.8 and 73.3 nC correspond to the straightening process of the middle, top and bottom joints in sequence. The corresponding optical photographs of every practical action state are illustrated in Fig. 4c as insets. It noted that a subtle difference exists between the actual transferred charge quantity and the characteristic value. This may be due to the imperfect random action of a finger, which cannot operate ideally to achieve a complete contacting and separating process. However, the tiny disparity has negligible influence in estimating the action of the finger due to the quantity sequence from largest to smallest in accordance with the bottom, middle, and top. Hence, as shown in Fig. 4d, a rising step of 112.2 nC is deduced to be the bending of the three joints together, consistent with the optical photograph of bending all joints. Also, according to the principle discussed above, the following declining steps of 10.5, 27.4, and 74.3 nC coincide with the unbending operation of the top, middle, and bottom joint, respectively, with the photograph of the actual operation state inserted in the appropriate locations.

## Conclusions

In summary, TENGs to be combined with a finger are fabricated to form a self-powered sensor system. This sensor can transform the mechanical energy of the fingers into electrical signal with the advantages of low-cost, environmentally friendly, simple fabrication, and high output. A single TENG combined with a finger (with an area of 10.5 cm^2^) generated an output current of 7.8 μA, a transferred charge of 78 nC and an output voltage of 130 V. Then, the transferred charges and voltage remain balanced while the current is enhanced with the increase of the operation frequency. The output power can be optimized when the external resistance is at approximately 30 MΩ. Finally, a self-powered sensor system is fabricated with TENGs for different areas attached to different joints to detect the random motions of a finger. The transferred charge quantity is chosen as a characteristic parameter to deduce the complicated motions of a finger. This device will find applications in many fields, such as electronic skins, artificial intelligence and robotics.

## Methods

Fabrication and measurement of TENGs: A 30 μm-thick FEP and aluminum foil were adopted to be the friction materials. Aluminum foil was adhered onto the surface of the finger acting as one material layer. Then the FEP film (size of 1.5 cm × 2 cm, 1.5 cm × 3 cm, and 1.5 cm × 7 cm) with the electrode of aluminum foil was adhered onto the substrate PET, working as another material layer. An SR570 (Stanford Research System) has been used to measure the short-circuit current. And the Keithley 6514 electrometer has been used to test transferred charge and open-circuit voltage.

## References

[1] Rim YS, Bae S-H, Chen H, De Marco N, Yang Y (2016). Recent Progress in Materials and Devices toward Printable and Flexible Sensors. Adv. Mater..

[2] Huang W (2015). A High-Capacitance Salt-Free Dielectric for Self-Healable, Printable, and Flexible Organic Field Effect Transistors and Chemical Sensor. Adv. Funct. Mater..

[3] Chortos A, Bao Z (2014). Skin-inspired electronic devices. Mater. Today.

[4] Gielen D, Boshell F, Saygin D (2016). Climate and energy challenges for materials science. Nature Mater.

[5] Kaygusuz K (2001). Renewable energy: Power for a sustainable future. Energy Explor. Exploit.

[6] Niu, S., Wang, X., Yi, F., Zhou, Y. S. & Wang, Z. L. A universal self-charging system driven by random biomechanical energy for sustainable operation of mobile electronics. *Nat. Commun*. **6** (2015). 10.1038/ncomms9975 PMC4682168 26656252

[7] Wang S, Xie Y, Niu S, Lin L, Wang ZL (2014). Freestanding Triboelectric-Layer-Based Nanogenerators for Harvesting Energy from a Moving Object or Human Motion in Contact and Non-contact Modes. Adv. Mater..

[8] Wang ZL (2013). Triboelectric Nanogenerators as New Energy Technology for Self-Powered Systems and as Active Mechanical and Chemical Sensors. Acs Nano.

[9] Fan F-R, Tian Z-Q, Wang ZL (2012). Flexible triboelectric generator!. Nano Energy.

[10] Kim J (2017). Research Update: Hybrid energy devices combining nanogenerators and energy storage systems for self-charging capability. Apl Materials.

[11] Fan F-R (2012). Transparent Triboelectric Nanogenerators and Self-Powered Pressure Sensors Based on Micropatterned Plastic Films. Nano Lett..

[12] Wang S, Lin L, Wang ZL (2012). Nanoscale Triboelectric-Effect-Enabled Energy Conversion for Sustainably Powering Portable Electronics. Nano Lett..

[13] Zhu G (2013). Linear-Grating Triboelectric Generator Based on Sliding Electrification. Nano Lett..

[14] Lee J-H (2016). All-in-one energy harvesting and storage devices. J. Mater. Chem. A.

[15] Wang ZL, Chen J, Lin L (2015). Progress in triboelectric nanogenerators as a new energy technology and self-powered sensors. Energy Environ. Sci..

[16] Zhu G (2014). A Shape-Adaptive Thin-Film-Based Approach for 50% High-Efficiency Energy Generation Through Micro-Grating Sliding Electrification. Adv. Mater..

[17] Wang S, Lin L, Wang ZL (2015). Triboelectric nanogenerators as self-powered active sensors. Nano Energy.

[18] Zhu G (2014). Harvesting water wave energy by asymmetric screening of electrostatic charges on a nanostructured hydrophobic thin-film surface. ACS nano.

[19] Xu L (2017). Integrated triboelectric nanogenerator array based on air-driven membrane structures for water wave energy harvesting. Nano Energy.

[20] Li X (2015). A self-powered system based on triboelectric nanogenerators and supercapacitors for metal corrosion prevention. J. Mater. Chem. A.

[21] He X (2016). A Highly Stretchable Fiber‐Based Triboelectric Nanogenerator for Self‐Powered Wearable Electronics. Adv. Funct. Mater..

[22] Zhao XJ, Zhu G, Fan YJ, Li HY, Wang ZL (2015). Triboelectric charging at the nanostructured solid/liquid interface for area-scalable wave energy conversion and its use in corrosion protection. ACS nano.

[23] Lin ZH, Cheng G, Lin L, Lee S, Wang ZL (2013). Water–Solid Surface Contact Electrification and its Use for Harvesting Liquid‐Wave Energy. Angew. Chem. Int. Ed..

[24] Hanson, M. A. *et al*. Body area sensor networks: Challenges and opportunities. *Computer* **42** (2009).

[25] Takamatsu S, Kobayashi T, Shibayama N, Miyake K, Itoh T (2012). Fabric pressure sensor array fabricated with die-coating and weaving techniques. Sens. Actuators, A.

[26] Cohn, G. *et al*. In *Proceedings of the 2012 ACM Conference on Ubiquitous Computing*. 99–102 (ACM).

[27] Yamada T (2011). A stretchable carbon nanotube strain sensor for human-motion detection. Nature Nanotech.

[28] Lee J (2014). A stretchable strain sensor based on a metal nanoparticle thin film for human motion detection. Nanoscale.

